# Predictors of Food and Physical Activity Tracking Among Young Adults

**DOI:** 10.1177/10901981231159679

**Published:** 2023-03-20

**Authors:** Erin K. O’Loughlin, Maryam Marashi, Catherine M. Sabiston, Kristen M. Lucibello, Marie-Pierre Sylvestre, Jennifer L. O’Loughlin

**Affiliations:** 1 Centre de recherche du centre hospitalier de l’Université de Montréal (CRCHUM), Montreal, Quebec, Canada; 2 University of Toronto, Toronto, Ontario, Canada; 3 Université de Montréal, Montreal, Quebec, Canada

**Keywords:** My Fitness Pal, food tracking, eating disorders, physical activity tracking, fitbit, longitudinal study, physical activity, young adults

## Abstract

**Background:**

Monitoring food intake and physical activity (PA) using tracking applications may support behavior change. However, few longitudinal studies identify the characteristics of young adults who track their behavior, findings that could be useful in designing tracking-related interventions. Our objective was to identify predictors of past-year food and PA tracking among young adults.

**Methods:**

Data were available for 676 young adults participating in the ongoing longitudinal Nicotine Dependence in Teens Study. Potential predictors were measured in 2017–2020 at age 31, and past-year food and PA tracking were measured in 2021–2022 at age 34. Each potential predictor was studied in a separate multivariable logistic regression model controlling for age, sex, and educational attainment.

**Results:**

One third (37%) of participants reported past-year PA tracking; 14% reported past-year food, and 10% reported both. Nine and 11 of 41 potential predictors were associated with food and PA tracking, respectively. Compensatory behaviors after overeating, trying to lose weight, self-report overweight, reporting a wide variety of exercise behaviors, and pressure to lose weight predicted both food and PA tracking.

**Conclusion:**

Food and PA tracking are relatively common among young adults. If the associations observed herein between compensatory behavior after overeating and tracking (among other observed associations) are replicated and found to be causal, caution may need to be exercised in making “blanket” recommendations to track food intake and/or PA to all young adults seeking behavior change.

## Introduction

Monitoring food intake and physical activity (PA) using tracking applications (i.e., apps) is encouraged by fitness coaches, dietitians, and clinicians to support behavior change (Berry et al., 2021; Boutelle & Kirschenbaum, 1998; Harvey et al., 2019). Food tracking comprises logging the types (e.g., food groups, macronutrient counts) or quantities (e.g., caloric intake) of foods consumed. PA tracking involves recording steps or distances walked, frequency and/or intensity of PA, and/or caloric burn of exercise sessions. Bandura (1998) suggest that self-monitoring is a key component of health behavior change that increases self-awareness of behavioral patterns, which can then translate into intentional change in behaviors targeted. Tracking can be undertaken with social support through coaching (e.g., health and fitness coaches, personal trainers, dieticians, nutritionists) or with purchased automated apps which provide support with goal setting and evaluation of progress (Berry et al., 2021). Weight loss is the most frequently reported reason for food and PA tracking (Chen et al., 2017; Kinney et al., 2019). Other reasons include to increase PA, develop healthier eating patterns, manage diabetes or other health conditions, improve workouts, achieve a fitness or sporting goal, and monitor sleep (Chen et al., 2017; Kinney et al., 2019).

Although a popular approach to promote behavior change (Berry et al., 2021; Boutelle & Kirschenbaum, 1998; Harvey et al., 2019), there is growing concern that food and PA tracking may have unintended negative effects on health such as increasing the risk of disordered eating, unhealthy body-related compensatory behaviors, compulsive exercise symptoms, and body dissatisfaction (Embacher Martin et al., 2018; Hahn, Sonneville, et al., 2021; Plateau et al., 2018; Simpson & Mazzeo, 2017). Several factors may underpin these negative effects. First, food and PA tracking apps usually provide users with instant feedback on their “progress” (or lack thereof) toward goals or incentives that are either self-set or recommended by the app or a coach. Feedback typically informs users visually whether they are at, under, or above their allotted caloric, macronutrient (e.g., carbohydrate, dietary fat, protein), step (e.g., 10,000 steps per day), or activity (i.e., 150 min per week) targets. Mismatches between attained behavior and expectations or hopes in terms of targeted behavior could elicit feelings of shame, guilt, stress and/or anxiety in users (Berry et al., 2020), and/or perpetuate perfectionist tendencies (Helms et al., 2019; Levinson et al., 2017; Linardon & Messer, 2019).

Second, relying on automated feedback rather than individual personal feedback may distance a person from intuitive eating (Tylka & Wilcox, 2006) and compassionate movement (i.e., focusing on the intrinsic value, process, and enjoyment of bodily movement, rather than quantifiable metrics such as “high intensity,” vigilant self-monitoring and the pursuit of outcome-based goals such as weight loss; Pila et al., 2019), both of which relate to positive health outcomes.

Finally, although food and PA tracking are “individualized,” many apps use population-based algorithms to set targets for PA and calorie intake which bypass important individual characteristics such as dieting history, exercise levels/history, disease history, personal preferences, body image, sleep quantity and quality, and life stress. Given that personalized mobile health interventions based on an individual’s unique psychological profile and life history increase the likelihood of successful health behavior change (Walsh & Groarke, 2019), app-based recommendations may not be attainable or even appropriate for some individuals.

Growing evidence in fact supports that tracking may not be appropriate for all people (Embacher Martin et al., 2018; Hahn, Sonneville, et al., 2021; Helms et al., 2019; Levinson et al., 2017; Linardon & Messer, 2019; Plateau et al., 2018; Simpson & Mazzeo, 2017), and researchers are now beginning to document the characteristics of people who engage in tracking, in addition to exploring whether tracking is harmful or helpful. Online Supplementary Tables 1 and 2 summarize known correlates and predictors of food and PA tracking across studies. Briefly, in cross-sectional studies, correlates of both food and PA tracking reported to date for young adults include female sex, higher body mass index (BMI) (Hahn, Sonneville, et al., 2021), unhealthy weight-control behaviors (i.e., fasting, using food substitutes, taking diet pills, purging), conventional weight-control behaviors, (i.e., increasing fruit and vegetable consumption, eating fewer high-fat foods and sweets), and conventional muscle-building behaviors (i.e., changes to eating or exercise to promote muscle growth; Hahn, Hazzard, Loth, et al., 2022). In a recent longitudinal study focused on weight- and muscle-related measures and behaviors, Hahn, Hazzard, Larson, et al. (2022) identified predictors of food and PA tracking in a sample of college-age adults. After adjusting for sociodemographic variables, BMI ≥ 85th percentile during adolescence predicted food and PA tracking 8 years later. In further modeling that also adjusted for BMI, body dissatisfaction, unhealthy muscle-building behaviors (i.e., described by the authors of the study as use of protein powders, steroids, or other muscle-building substances), and conventional muscle-building behaviors in adolescence predicted both forms of tracking (Hahn, Hazzard, Larson, et al., 2022). Apart from Hahn, Hazzard, Larson, et al. (2022), no other longitudinal studies have identified predictors of food and PA tracking behavior.

Based on our review, we identified three important gaps in this literature. First, the range of potential correlates or predictors of tracking tends to be limited, obviating identification of a range of sociodemographic, psychological, emotional, and behavioral predictors. Broadening the scope of potential predictors could capture, for example, that it is the intent underpinning tracking rather than the actual monitoring that is relevant in the relationship between tracking and disordered eating or PA. Second, identifying predictors in longitudinal studies is key in terms of replicating findings from cross-sectional studies in a stronger study design. Finally, most of the extant literature has focused on adolescents (i.e., ages 10–19) or college-age adults (i.e., ages 19–25). Few studies explore tracking behaviors in persons ages 25–35. Differences in developmental stage and life situations across age groups may be germane to the relationship between tracking behavior and disordered eating and exercise behaviors, and therefore, it is important to expand the study of tracking behaviors to include adults across the lifespan.

The objective of this study was therefore to identify predictors of food and PA tracking in a population-based sample of young adults in their early thirties, from among a broad range of diverse potential predictors in a longitudinal study design. The findings could be useful in the design and targeting of tracking-related interventions and they may help identify population subgroups in which tracking could be unhelpful in behavior change or even detrimental to health.

## Method

Data were drawn from the Nicotine Dependence in Teens study (NDIT), an ongoing 22-year longitudinal study that aimed to describe the natural course of nicotine dependence in youth, but also collected data on a wide range of sociodemographic, psychosocial, lifestyle, and health-related variables. More information on the NDIT study, including its study design and copies of the questionnaires can be found at https://www. https://www.celphie.ca/ndit. Relevant to this study, data were available for 799 participants in NDIT cycle 23 (mean (*SD*) age = 30.5 (1.0)) and 722 participants in NDIT study cycle 24 (mean (*SD*) age = 33.6 (0.5)).

A total of 676 participants (85% of 799 participants in cycle 23) provided data in both cycles 23 and 24 and were retained for analysis in this study. Data on factors potentially associated with tracking were drawn from cycle 23 (which is considered to be “baseline” in this study), and data on the “outcomes” (i.e., past-year food-tracking; past-year PA tracking) were drawn from cycle 24. In addition to factors potentially associated with tracking measured in cycle 23, we also studied several time-invariant exposures (e.g., age, sex, born in Canada, mother university-educated) which were measured in earlier data collection cycles.

NDIT was approved by ethics committees at the Montreal Department of Public Health, McGill University and the University of Montréal (2007–2384, 2017–6895, ND06.087). Informed parental consent was obtained in cycle 1. Participants (who had all attained legal age) provided consent in the post high school data collections.

### Study Variables

*Past-year food tracking* was measured by: “In the past 12 months, how often did you use an app to track your food intake (My Fitness Pal, Carbon diet coach, Noom, etc.)” Response choices included never, less than once a month, 1–3 times a month, 1–6 times a week, and everyday. For analyses, responses were recoded as no (never) or yes (all other responses).

*Past-year PA tracking* was measured by: “In the past 12 months, how often did you wear a fitness device or use a smartphone app that monitors your PA?” Response choices included never, less than once a month, 1–3 times a month, 1–6 times a week, and everyday. For analyses, responses were recoded as no (never) or yes (all other responses).

Potential predictors were selected based on factors known to be associated with food and/or PA tracking and PA in general (Dowda et al., 2003; Martins et al., 2017; Quick et al., 2013; Sleddens et al., 2015; Stevenson, 2017) (see online Supplementary Tables 1 and 2), as well as on the availability of data in the NDIT study. Potential predictors included five *sociodemographic indicators* (i.e., age, sex, participant attended university (yes, no), household income (<30,000, 30,000 < 99,000, ≥100,000 CAD), employed (yes, no)); nine *lifestyle behaviors* (i.e., smoked cigarettes in past-year (no, yes), binge drank in past-year (never, less than monthly, monthly, weekly, daily) meets moderate to vigorous PA (MVPA) guidelines (no, yes), team sports in past-year (no, yes), sleep quality (poor, fair, good, very good, excellent) as well as four indicators of eating behavior (i.e., frequency of overeating (never, rarely, sometimes, often, very often), total fruits and vegetables per day, junk food per day, compensatory behavior after overeating)), three *weight-related indicators* (i.e., BMI, self-report overweight (no, yes), trying to lose weight (no, yes)), 15 *psychological indicators* (i.e., depressive symptoms, self-esteem, perceived daily stress (not at all, not very, a bit, quite, extremely stressful), *body-related emotions* of shame, guilt, authentic pride, envy, embarrassment and hubristic pride, *PA behavior regulations* including amotivation, external introjected, identified and intrinsic, and variety of exercise behaviors), four indicators pertaining to *family/peer social influence* (i.e., pressure to engage in PA, pressure to lose and pressure to gain weight (never, rarely, sometimes, often, always), negative comments about weight (never, rarely, sometimes, often, always)), and finally five *health-related indicators* (self-rated health and self-rated mental health (poor, fair, good, very good, excellent), diagnosed anxiety disorder (no, yes), diagnosed mood disorder (no, yes), diagnosed eating disorder (no, yes)).

Supplementary Table 4 describes each variable in detail including the questionnaire items used to collect data on the variable, response options, re-coding of response options for analysis, Cronbach’s alpha for scales, and references.

### Data Analysis

We conducted descriptive analyses to check distributions, identify missing data and outliers, to compute means and standard deviations (*SD*) for continuous variables and proportions for categorical variables, and to compare participants retained and not retained in the analysis. With the exception of BMI (14% of participants were missing data on BMI), data were missing for 0–8% of participants for all other variables (see online Supplementary Table 4).

We investigated each potential predictor in a complete case analysis as an independent study that addressed a single hypothesis (Bender & Lange, 2001). We first examined the associations between each potential predictor and past-year food and PA tracking use in cross-tabulations. Then only two models were estimated for each predictor variable—an unadjusted logistic regression model and a multivariable model adjusting for age, sex, and educational attainment. Continuous variables were retained as continuous in both the unadjusted and adjusted logistic regression models. We did not include BMI as a potential confounder in the adjusted models because it may be on the causal pathway between the “exposures” (i.e., predictors) and the “outcomes” (i.e., past-year food and activity tracking). Inclusion of a mediator in the models could incorrectly attenuate the associations (Schisterman et al., 2009).

We did not estimate a model including all predictors because adjustment by variables that are not confounders of the association between the exposure and the outcome (but rather colliders or mediators) could bias the estimated coefficient (Schisterman et al., 2009; Westreich & Greenland, 2013). We concluded that a variable was associated with past-year tracking if the confidence interval for the estimate excluded the null value of 1.0 (Poole, 2001).

Data were analyzed using SPSS version 20.0 (released 2011, SPSS Statistics for Windows; IBM Corp). All statistical tests were two-sided, with the significance level set at 0.05.

## Results

The mean (*SD*) age of the 676 participants retained for analysis was 30.5 (1.0) in Cycle 23 and 33.6 (0.5) years in Cycle 24, 41% were male, 95% were born in Canada, and 34% reported that French was the language spoken at home in NDIT study Cycle 1. In Cycle 24, 21% had an annual household income <US$50,000 CAN, 15% lived alone, 51% lived with children, 83% reported being employed, 60% had attended university and 36% met MVPA guidelines.

Comparison of the baseline (i.e., cycle 23) characteristics of participants retained and not retained in this analysis (Table 1) suggests that lower proportions of participants retained were male (41% vs. 60%) and French-speaking (29% vs. 39%).

**Table 1.** Characteristics of Participants Retained and Not Retained for Analysis at Baseline in Cycle 23, NDIT Study 2017–2021.

Characteristic^a^	Retained (*n* = 676)	Not retained (*n* = 123)
Age, y, mean (*SD*)	30.5 (1.0)	31.0 (1.1)
Male, %	41.1	60.2
Born in Canada, %	94.3	90.7
Mother university-educated, %	54.4	50.0
French-speaking, %	29.2	39.0
Walking, minutes/week, median (IQR)	175 (60, 350)	200 (70, 420)
Met MVPA guidelines, %	48.9	53.0

*Note.* NDIT = Nicotine Dependence in Teens; IQR = interquartile range; y = years; MVPA = moderate-to-vigorous physical activity.

a All data were drawn from Cycle 23.

Thirty-seven percent of participants (*n*=249) reported past-year PA tracking, and 14% (*n* = 91) reported past-year food tracking. Sixty-five of the 91 participants who reported past-year food tracking (i.e., 71.0%), also reported past-year PA tracking (i.e., 10.0% of the 676 participants retained for analysis). Only 26.0% of PA trackers (i.e., 65 of 249) also reported food tracking.

### Predictors of Past-Year PA Tracking

Eleven of 41 potential predictors were associated with past-year PA tracking (Table 2). Three of five sociodemographic variables were retained including attended university, higher household income, and being employed. Of the nine lifestyle behaviors, meets MVPA guidelines and reporting more compensatory behaviors after overeating were associated with PA tracking. Participants who self-reported overweight and those trying to lose weight were more likely to track PA. Only 2 of the 15 psychological indicators were associated, one of which pertained to PA behavior regulations (i.e., higher identified behavior regulations). Persons who reported engaging in a wide variety of exercise behaviors were more likely to track PA. One of the four family and peer influence indicators (i.e., pressure to lose weight) was a predictor. Finally, participants with a diagnosed eating disorder were 5.3 times more likely than those without an eating disorder to report PA tracking.

**Table 2.** Odds Ratio (OR) and 95% Confidence Interval (CI) for the Crude and Adjusted Association Between Potential Predictors and Past-Year Food and Physical Activity Tracking Among Young Adults, NDIT Study 2017–2021 (n = 676).

	*n* ^d^	Past-year food tracking			Past-year PA tracking		
Potential predictors		%	OR_crude_ (95% CI)	OR_adj_ (95% CI)^a^	%	OR_crude_ (95% CI)	OR_adj_ (95% CI) ^a^
Sociodemographic indicators							
Age, y^b^			1.0 [0.8, 1.2]	1.0 [0.8, 1.3]		1.0 [0.8, 1.1]	1.0 [0.8, 1.2]
<30.1	264	14.0			36.0		
≥30.1 < 30.8	182	12.1			39.0		
≥30.8	230	13.9			36.1		
Sex							
Male	279	9.0	ref	ref	32.3	ref	ref
Female	397	16.6	**2.0 [1.2, 3.3]**	**2.0 [1.2, 3.2]**	40.1	1.4 [1.0, 1.9]	1.4 [1.0, 1.9]
Participant attended university							
No	253	10.3	ref	ref	25.7	ref	ref
Yes	420	15.2	1.6 [1.0, 2.6]	1.6 [1.0, 2.6]	43.6	**2.2 [1.6, 3.1]**	**2.3 [1.6, 3.2]**
Household income, CAD$							
<30,000	70	11.4	ref	ref	20.0	**ref**	**ref**
30,000 < 99,000	363	12.4	1.1 [0.5, 2.4]	1.2 [0.5, 2.7]	35.8	**2.2 [1.2, 4.2]**	**2.2 [1.2, 4.3]**
≥100,000	209	15.8	1.5 [0.6, 3.3]	1.5 [0.7, 4.0]	46.4	**3.5 [1.8, 6.6]**	**3.2 [1.6, 6.2]**
Employed							
No	99	11.1	ref	ref	24.2	ref	ref
Yes	570	13.9	1.3 [0.7, 2.5]	1.4 [0.7, 2.8]	38.9	**2.0 [1.2, 3.3]**	**2.1 [1.3, 3.5]**
Lifestyle behaviors							
Smoked cigarettes in past 12 months							
No	465	14.4	ref	ref	38.1	ref	ref
Yes	211	11.4	0.8 [0.5, 1.3]	0.9 [0.5, 1.4]	34.1	0.8 [0.6, 1.2]	1.0 [0.7, 1.4]
Binge drank in past-year^b^			0.9 [0.8, 1.2]	1.0 [0.8, 1.3]		1.1 [0.9, 1.3]	1.2 [1.0, 1.4]
Never	229	12.2			31.9		
Less than weekly	381	14.4			40.2		
Weekly/daily	59	10.2			35.6		
Meets MVPA guidelines							
No	338	12.7	ref	ref	31.1	ref	ref
Yes	324	14.2	1.1 [0.7, 1.8]	1.3 [0.9, 2.1]	43.5	**1.7 [1.2, 2.4]**	**1.9 [1.4, 2.7]**
Team sports in past-year							
No	515	14.2	ref	ref	35.5	ref	ref
Yes	155	11.6	0.8 [0.5, 1.4]	0.9 [0.5, 1.6]	41.3	1.3 [0.9, 1.8]	1.4 [0.9, 2.0]
Sleep quality^a^			1.0 [0.8, 1.2]	1.0 [0.8, 1.2]		1.0 [0.8, 1.1]	0.9 [0.8, 1.1]
Poor/fair	266	15.8			38.7		
Good	214	7.5			34.6		
Very good/excellent	194	16.5			36.6		
Eating behavior							
Frequency overeating^b^			**1.4 [1.2, 1.7]**	**1.4 [1.1, 1.8]**		1.1 [1.0, 1.3]	1.2 [1.0, 1.3]
Never/rarely	276	10.1			31.5		
Sometimes	265	15.1			42.3		
Often/very often	114	18.4			38.6		
Total fruits and vegetable/day^b^			0.9 [0.9, 1.1]	1.0 [0.9, 1.1]		1.1 [1.0, 1.1]	1.1 [1.0, 1.1]
<3.8	346	12.7			34.7		
≥3.8	282	13.8			40.1		
Junk food/day^b^			0.8 [0.6, 1.1]	0.9 [0.7, 1.3]		**0.7 (0.6, 0.9]**	0.8 [0.6, 1.0]
<0.7	341	13.2			32.7		
≥0.7	278	12.9			41.3		
Compensatory behavior after overeating^b^			**1.8 [1.3, 2.4]**	**1.7 [1.2, 2.4]**		**1.5 [1.2, 2.0]**	**1.6 [1.3, 2.0]**
<2.8	235	9.4			29.4		
≥2.8	331	18.7			44.1		
Weight-related indicators							
BMI ^b^			1.1 [1.0, 1.1]	1.1 [1.0, 1.1]		1.0 [1.0, 1.1]	1.0 [1.0, 1.1]
<18.5	12	0.0			16.7		
18.5 < 24.9	286	11.9			35.0		
≥25.0	281	17.4			39.5		
Self-report overweight							
No	368	10.3	ref	ref	33.4	ref	ref
Yes	306	17.0	**1.8 [1.1, 2.8]**	**1.9 [1.2, 2.9]**	41.0	1.4 [1.0, 1.9]	**1.5 [1.1, 2.1]**
Trying to lose weight							
No	365	8.2	ref	ref	32.0	ref	ref
Yes	306	19.6	**2.8 [1.7, 4.6]**	**2.9 1.8, 4.6]**	43.5	**1.7 [1.2, 2.3]**	**1.9 [1.4, 2.6]**
Psychological indicators							
Depressive symptoms^b^			1.0 [1.0, 1.0]	1.0 [1.0, 1.0]		1.0 [1.0, 1.1]	1.0 [1.0, 1.1]
0.0–8.0	189	13.8			35.4		
9.0–12.0	249	13.7			37.8		
≥13.0	223	13.0			36.8		
Self-esteem^b^			1.3 [0.8, 2.0]	1.3 [0.8, 2.0]		0.9 [0.7, 1.2]	0.9 [0.6, 1.2]
0.0–3.0	284	12.0			37.3		
3.1–3.4	168	11.9			36.3		
≥ 3.5	221	16.3			36.2		
Daily stress^b^			1.2 [1.0, 1.6]	1.2 [0.9, 1.5]		**1.3 [1.1, 1.5]**	1.3 [1.0, 1.5]
Not at all/not very stressful	157	10.8			29.3		
A bit stressful	327	13.1			37.6		
Quite/extremely stressful	190	15.8			41.6		
Body-related emotions							
Body-related shame^b^			1.3 [1.0, 1.7]	1.3 [1.0, 1.7]		1.1 [0.9, 1.4]	1.1 [0.9, 1.4]
<1.8	221	11.8			32.6		
≥1.8	408	13.7			39.5		
Body-related guilt^b^			1.2 [1.0, 1.5]	1.2 [1.0, 1.5]		1.1 [0.9, 1.3]	1.1 [0.9, 1.3]
<1.8	280	13.2			36.1		
≥1.8	349	12.9			37.8		
Body-related authentic pride^b^			1.1 [0.9, 1.4]	1.1 [0.9, 1.4]		1.2 [1.0, 1.5]	1.2 [1.0, 1.5]
<2.3	277	11.9			32.1		
≥2.3	352	13.9			40.9		
Body-related envy^b^			1.3 [1.0, 1.6]	1.2 [1.0, 1.6]		1.2 [1.0, 1.4]	1.2 [1.0, 1.4]
<2.0	280	11.4			31.4		
≥2.0	349	14.3			41.5		
Body-related embarrassment^b^			**1.3 [1.1, 1.7]**	**1.3 [1.1, 1.7]**		1.1 [0.9, 1.4]	1.2 [1.0, 1.4]
<1.8	299	11.4			31.8		
≥1.8	330	14.5			41.8		
Body-related hubristic pride^b^			1.0 [0.7, 1.3]	1.0 [0.7, 1.3]		1.1 [0.9, 1.3]	1.1 [0.9, 1.3]
<2.3	271	14.0			35.8		
≥2.3	358	12.3			38.0		
PA behavior regulation^b^							
Amotivation			0.8 [0.4, 1.3]	0.9 [0.5, 1.5]		0.9 [0.6, 1.2]	1.0 [0.7, 1.4]
No	496	13.9			38.5		
Yes	133	9.8			31.6		
External			1.2 [0.9, 1.7]	1.2 [0.9, 1.7]		1.2 [1.0, 1.6]	1.2 [1.0, 1.5]
<0.3	272	10.3			33.8		
≥0.3	357	15.1			39.5		
Introjected			**1.4 [1.2, 1.8]**	**1.4 [1.2, 1.8]**		**1.3 [1.1, 1.4]**	1.2 [1.0, 1.4]
<1.8	274	8.8			31.4		
≥1.8	355	16.3			41.4		
Identified			1.4 [1.0, 1.8]	1.4 [1.0 1.8]		**1.4 [1.2, 1.7]**	**1.3 [1.1, 1.6]**
<2.8	280	10.7			32.5		
≥2.8	349	14.9			40.7		
Intrinsic			1.2 [0.9, 1.5]	1.2 [0.9, 1.5]		1.2 [1.0, 1.4]	1.1 [1.0, 1.3)
<2.5	278	11.5			33.8		
≥2.5	351	14.2			39.6		
Variety of exercise behaviors^b^			**1.4 [1.2, 1.6]**	**1.4 [1.2, 1.7]**		**1.3 [1.1, 1.4]**	**1.3 [1.1, 1.4]**
<3.9	277	10.1			28.2		
≥3.9	394	15.5			42.6		
Family/peer social influence							
Pressure to engage in PA^b^			1.0 [0.8, 1.3]	1.0 [0.8, 1.3]		1.0 [0.8, 1.2]	1.0 [0.9, 1.2]
<2.8	249	14.9			37.8		
≥2.8	414	12.6			35.5		
Pressure to lose weight^b,c^			**1.4 [1.2, 1.7]**	**1.5 [1.2, 1.8]**		1.1 [0.9, 1.3]	**1.2 [1.0, 1.4]**
Never/rarely	475	10.7			4.7		
Sometimes	129	17.1			45.0		
Often/always	66	25.8			34.8		
Pressure to gain weight^b,c^			0.7 [0.5, 1.0]	0.7 [0.5, 1.0]		0.8 [0.7, 1.0]	0.8 [0.7, 1.0]
Never/rarely	573	14.7			38.6		
Sometimes	77	7.8			20.8		
Often/always	21	0.0			38.1		
Negative comments about weight^b,c^			1.2 [1.0, 1.5]	1.2 [1.0, 1.6]		0.9 [0.7, 1.0]	0.9 [0.7, 1.1]
Never/rarely	497	12.7			38.0		
Sometimes	132	12.1			34.8		
Often/always	43	25.6			25.6		
Health-related indicators							
Self-rated health^b^			1.1 [0.8, 1.4]	1.0 [0.8, 1.3]		1.2 [1.0, 1.4]	1.1 [0.9, 1.3]
Poor/fair	137	14.6			30.7		
Good	299	11.0			33.8		
Very good/excellent	237	15.6			43.9		
Self-rated mental health^b^			1.0 [0.9, 1.1]	1.0 [0.9, 1.1]		1.1 [0.9, 1.2]	1.0 [0.9, 1.2]
Poor/fair	140	10.7			37.1		
Good	248	12.1			33.1		
Very good/excellent	284	15.8			39.8		
Diagnosed anxiety disorder							
No	534	12.4	ref	ref	35.8	ref	ref
Yes	96	17.7	1.5 [0.9, 2.7]	1.4 [0.8, 2.6]	44.8	1.5 [0.9, 2.3]	1.5 [0.9, 2.3]
Diagnosed mood disorder							
No	558	13.1	ref	ref	37.1	ref	ref
Yes	72	14.0	1.1 [0.5, 2.2]	1.1 [0.5, 2.0]	37.5	1.0 [0.6, 1.7]	1.0 [0.6, 1.7]
Diagnosed eating disorder							
No	620	12.7	ref	ref	36.5	ref	ref
Yes	10	40.0	**4.6 [1.3, 16.6]**	3.3 [0.9, 12.3]	80.0	**7.0 [1.5, 33.2]**	**5.3 [1.1, 25.9]**

*Note.* Bolded indicates that the CIs excluded the null value of 1.0. NDIT = Nicotine Dependence in Teens; PA = physical activity; y = years; CAD = Canadian currency; MVPA = moderate-vigorous physical activity; BMI = body mass index.

a Model adjusted for age, sex, and participant education. ^b^ Variable was retained as continuous in both the unadjusted and adjusted logistic regression models. ^c^ In past 2 years. ^d^ Totals differ due to missing data.

### Predictors of Past-Year Food Tracking

Nine of 41 potential predictors were associated with past-year food tracking (Table 2). Among the five sociodemographic variables, only sex was retained—females were twice as likely to report food tracking. Only two of nine lifestyle-related variables were related to food tracking including a higher frequency of overeating and reporting more compensatory behaviors after overeating. Two of the three weight-related indicators were retained—participants who self-reported overweight were 1.9 times more likely to track food intake, and those trying to lose weight were almost three times more likely. Three of 15 psychological indicators predicted food tracking. The odds ratio for body-related embarrassment was 1.3 indicating that for each unit increase in body-related embarrassment, participants were 30% more likely to food track. Of the five indicators of PA behavior regulations, only higher introjected regulation predicted food tracking. Persons who participated in a wide variety of exercise behaviors were more likely to food track as well as those who felt pressure to lose weight. Finally, none of the health-related indicators was associated with past-year food tracking. In unadjusted analyses, participants with a diagnosed eating disorder were 4.6 times more likely to food track. However, after adjustment for covariates, the 95% confidence intervals encompassed the null, although the estimate remained indicative of an association.

Because BMI was missing for 14% of participants, we conducted sensitivity analyses to examine the associations between BMI and both PA and food tracking using BMI computed based on height and weight data collected in the self-report questionnaires (for which there were no missing data). The results were similar to those for measured BMI.

## Discussion

Aligned with (Hahn, Hazzard, Larson, et al., 2022; Hahn, Hazzard, Loth, et al., 2022; Hahn, Sonneville, et al., 2021), this study suggests that PA tracking is more prevalent than food tracking—37% of participants reported PA tracking, 14% reported food and 10.0% reported both. The predictors of tracking identified (i.e., diagnosis of an eating disorder, several weight-related indicators, PA, eating behaviors, body-related embarrassment, PA behavior regulation, sex, education, income, employment) are consistent with both previous research and theoretical tenets. These findings help identify individuals more likely to track their food and PA behaviors and they may inform the targets and content of tracking-related interventions for young adults in their thirties.

Among our more important results and reflective of Hahn, Hazzard, Larson, et al. (2022) who reported that unhealthy weight-control behaviors predicted food and PA tracking in emerging adulthood, those with an eating disorder in this study were more likely to engage in self-monitoring behaviors. Because NDIT did not measure tracking behaviors prior to cycle 24, it is unclear whether tracking was a relatively new or a long-standing behavior at age 34. It is possible that health professional counseling related to an eating disorder diagnosis included recommendations to monitor food and/or PA tracking. It is also possible that the factors underpinning the illness such as perfectionist tendencies, led to an increased pre-occupation with self-monitoring. This association warrants further research to assess alternative explanations of its underpinnings.

Two of the three weight-related indicators (i.e., self-report overweight, trying to lose weight) predicted both food and PA tracking. Aligned with earlier reports that weight loss is a common reason for tracking (Chen et al., 2017; Kinney et al., 2019), weight-related indicators may drive self-monitoring decisions. Reflective of the tenets of the *Tripartite Influence Model of Body Image* (i.e., that peers, family, and the media have direct effects on body image dissatisfaction and subsequent eating behaviors with mediators such as internalization of societal ideals of appearance and heightened appearance comparison tendencies connecting influences on disturbed body image with eating pathology (Thompson et al., 1999)), social and societal pressure to lose weight were also associated with both tracking behaviors in this study. Future studies will need to assess whether the *Tripartite Model* is applicable in the context of food and PA tracking.

Body-related embarrassment (i.e., a negative self-conscious emotion in contexts where the body’s appearance or function is on display) predicted food tracking. An antecedent of body-related embarrassment includes fear of negative evaluation by others (Vani et al., 2020), which aligns with our finding that social and societal pressures to lose and/or gain weight predict food-tracking behavior. Food tracking may function as a body image coping strategy used to relieve feelings of appearance-related embarrassment associated with social pressures or negative comments about a person’s body weight (Cash et al., 2005).

Participants who met MVPA guidelines were more likely to engage in PA tracking, suggestive that engaging in adequate levels of PA prompts PA tracking to confirm that PA targets are attained. The reverse (i.e., that PA tracking causes attainment of MVPA guidelines) is also possible, but not ascertainable in NDIT because of the difficulty of establishing the timeline of tracking onset.

Overeating predicted food-tracking behavior, and compensatory behaviors after overeating (e.g., purging, skipping meals, eating more fruits and vegetables, exercising) predicted both forms of tracking. This may reflect that some individuals who are planning to diet engage in overconsumption of hyperpalatable foods in anticipation of deprivation in food intake (Urbszat et al., 2002) or alternatively, it may represent the automatic and unplanned eating behaviors that prompt attempts at self-regulation through tracking behavior. Whatever the underpinnings, compensatory behaviors tend to be maladaptive and therefore this association warrants further examination in longitudinal studies in which the timing of exposure and outcomes is well-delineated.

Supportive of cross-sectional studies (e.g., Nuss & Li, 2021; E. K. O’Loughlin et al., 2022), identified regulation (i.e., when the motivation underpinning a behavior is based on values and to some extent internal (Ryan & Deci, 2000) predicted PA tracking. Identified regulation for PA is associated with the desire to maintain optimal health, which may be a reason to monitor PA levels (E. K. O’Loughlin et al., 2022; Teixeira et al., 2012). Activity tracking has also been related to increases in empowerment, confidence in training programs and motivation to achieve PA goals, all of which may increase self-determined motivations such as identified regulation (Duus et al., 2018; Karapanos et al., 2016; Little, 2017; E. K. O’Loughlin et al., 2022).

Introjected regulation (i.e., partially internalized motivation resulting in behavior to avoid shame and guilt) predicted food tracking. Previous studies have linked food tracking to appearance-related factors such as wanting to lose weight (e.g., Messer et al., 2021), which aligns with our results that all three weight-related factors predicted food tracking. Future research should identify subgroups of trackers who use tracking for adaptive versus maladaptive reasons (such as guilt and shame).

Higher education, income, and being employed predicted PA tracking. This could reflect higher access to resources (e.g., higher levels of knowledge, disposable income, access to professional counseling) in these subgroups which allowed them to perceive the health advantages of tracking, purchase equipment, and integrate this behavior into their lifestyles.

Finally, aligned with research showing that females are more likely to engage in dieting and weight-control behaviors (Kiefer et al., 2005; Wardle et al., 2004), female sex predicted food tracking in this study.

Inconsistent with previous studies (Hahn, Hazzard, Larson, et al., 2022; Hahn, Sonneville, et al., 2021; Plateau et al., 2018; Romano et al., 2018), BMI did not predict either tracking behavior. Similarly, despite the well-established link between alcohol use and PA in young adults (Dodge et al., 2017), binge drinking did not predict either form of tracking. Co-occurrence of behaviors indicative of eating disorders and binge drinking (e.g., skipping meals, reducing food intake, excessively exercising to prevent weight gain associated with alcohol consumption) has been coined “drunkorexia” to represent this phenomenon (Eisenberg & Fitz, 2014). Finally, although body-related guilt, shame, and envy are associated with restrictive eating (Solomon-Krakus et al., 2022), body-related embarrassment was the only self-conscious emotion that predicted tracking behavior in this study. Shame, guilt, and envy may play a more proximal role in behavior and the 3–4 year gap between data collection cycles in this study may have precluded their detection.

Strengths of the study include its longitudinal design and inclusion of a broad range of potential predictors. However, because many predictors of tracking were identified, we discussed categories of predictors (e.g., weight-related variables) rather than discussing each predictor in-depth. Although at inception, NDIT participants resembled those of same-age participants in a provincially-representative survey (J. O’Loughlin et al., 2015; Paradis et al., 2003), loss-to-follow-up since inception may have limited the generalizability of the findings. Selection bias due to loss-to-follow-up since baseline (i.e., cycle 23) in this study, misclassification in self-report data, and residual confounding may have biased the estimates. Given that 41 separate associations were examined, some statistically significant findings may be attributable to chance, although our findings generally align with those of previous cross-sectional studies as well as with the only longitudinal study reported to date (see online Supplementary Tables 1 and 2). We did not distinguish between different types of tracking apps and finally, we did not measure reasons for or timing of the onset of tracking.

## Conclusion

Food and PA tracking were relatively common in this population-based sample of young adults, and numerous predictors of food and PA tracking were identified from among a wide range of diverse potential predictors. Supportive of concerns about possible negative health effects of tracking, we detected associations between both food and PA tracking and (a) compensatory behavior after overeating (i.e., a maladaptive behavior) and (b) eating disorders. However, some positive associations were found (e.g., meeting MVPA guidelines and PA tracking), which indicate that some forms of tracking may be appropriate for some subgroups. If replicated and found to be causal, caution should be exercised in making “blanket” recommendations to track food intake and/or PA to all young adults seeking behavior change.

## Supplemental Material

sj-docx-1-heb-10.1177_10901981231159679 – Supplemental material for Predictors of Food and Physical Activity Tracking Among Young Adults Supplemental material, sj-docx-1-heb-10.1177_10901981231159679 for Predictors of Food and Physical Activity Tracking Among Young Adults by Erin K. O’Loughlin, Maryam Marashi, Catherine M. Sabiston, Kristen M. Lucibello, Marie-Pierre Sylvestre and Jennifer L. O’Loughlin in Health Education & Behavior
