# Supplementary material for: Predictors of Food and Physical Activity Tracking Among Young Adults
Source: Health Educ Behav. 2023 Mar 20;50(5):647–57. doi: 10.1177/10901981231159679 (PMC10492434; doi:10.1177/10901981231159679)
Supplement: sj-docx-1-heb-10.1177_10901981231159679 – Supplemental material for Predictors of Food and Physical Activity Tracking Among Young Adults [file sj-docx-1-heb-10.1177_10901981231159679.docx]

**ONLINE SUPPLEMENTAL MATERIAL**

**Table S1.** Correlates of food and physical activity tracking identified in cross-sectional studies

| Correlate | Type of tracking | Source | Direction |
| --- | --- | --- | --- |
| *Socio-demographic* |  |  |  |
| Age^*^ | Food | Hahn et al., 2021a | - |
| BMI ≥ 30^†^ | Food | Hahn et al., 2021a | + |
| Female sex | Both | Hahn et al., 2021a | + |
| Female sex | Food | Embacher et al., 2018 | + |
| College education | Both | Hahn et al., 2021a | + |
| High socioeconomic status^*^ | Food | Hahn et al., 2021a | + |
| Latino^*^ | Physical activity | Hahn et al., 2021a | + |
| *Psychological* |  |  | + |
| Body dissatisfaction | Food | Embacher et al., 2018 | + |
| Eating disorder symptoms |  |  |  |
| Eating concern | Either or both^b^ | Plateau et al., 2018 | + |
| Eating concern | Food | Simpson and Mazzeo, 2017 | + |
| Weight concern | Either or both^b^ | Plateau et al., 2018 | + |
| Weight concern | Both | Simpson and Mazzeo, 2017 | + |
| Shape concern | Both | Simpson and Mazzeo, 2017 | + |
| *Behavioral* |  |  |  |
| Disordered weight-control^a^ | Both | Hahn et al., 2022a | + |
| Conventional weight-control^†a^ | Food | Hahn et al., 2022a | + |
| Conventional weight-control^a^ | Physical activity | Hahn et al., 2022a | + |
| Disordered muscle-building^a^ | Both | Hahn et al., 2022a | + |
| Conventional muscle-building^a^ | Both | Hahn et al., 2022a | + |
| Eating disorder symptoms |  |  |  |
| Dietary restraint | Either or both^b^ | Plateau et al., 2018 | + |
| Dietary restraint | Both | Simpson and Mazzeo, 2017 | + |
| Binge eating | Food | Simpson and Mazzeo, 2017 | + |
| Purging | Either or both^b^ | Plateau et al., 2018 | + |
| Purging | Food | Simpson and Mazzeo, 2017 | + |
| Compulsive exercise |  |  |  |
| Weight | Either or both^b^ | Plateau et al., 2018 | + |
| Mood | Either or both^b^ | Plateau et al., 2018 | + |

*Observed in males only

^†^Observed in females only

^a^Adjusted for behavior during adolescence

^b^Authors dichotomized data into participants who reported some form of tracking (i.e., food, activity, or both) and participants who reported no tracking

**Table S2.** Predictors of food and physical activity tracking in longitudinal studies

| Predictor | Type of tracking | Source | Direction |
| --- | --- | --- | --- |
| *Body weight* |  |  |  |
| BMI ≥ 85th percentile^†^ | Food | Hahn et al., 2021a | Risk |
| BMI ≥ 85th percentile* | Physical activity | Hahn et al., 2021a | Risk |
| *Psychological/behavioral* |  |  |  |
| Body dissatisfaction^a^ | Both | Hahn et al., 2021a | Risk |
| Disordered muscle-building in adolescence^†a^ | Food | Hahn et al., 2021a | Risk |
| Conventional muscle building in adolescence^*a^ | Both | Hahn et al., 2021a | Risk |

All models adjusted for race/ethnicity, age, socioeconomic status, and emerging adulthood educational/student status

*Observed in males only

^†^Observed in females only

^a^Adjusted for BMI

**Table S3.** Description of study variables including the questionnaire item(s) used to measure the variable, response choices, and re-coding for analysis.

| **Variable (reference)** | **Item(s)** | **Response choices** | **Recoding for analysis** |
| --- | --- | --- | --- |
| Today’s date | What is today’s date? | (Day) (month) (year) | Used to calculate age |
| Sex | Are you a boy/man/male or a girl/women/female? | Male, female |  |
| Participant attended university | How far have you gone in school? | Attended high school, but did not graduate; graduated high school; attended CEGEP, community/technical college, but did not graduate; graduated CEGEP, community/technical college; attended university (or teacher’s college), but did not graduate; graduated university with a Bachelor’s degree; graduated university with a Master’s degree; graduated university with a PhD/professional degree; Other (specify) | Yes (attended or graduated university), no (high school or less, post high school, CEGEP) |
| Household income | What is your best estimate of the total income, before taxes and deductions, of all household members from all sources in the past 12 months? | Less than $20,000, 20 000$ - 29 999$, 30 000$ - 39 999$, 40 000$ - 49 999$, 50 000$ - 59 999$, 60 000$ - 69 999$, 70 000$ - 79 999$, 80 000$ - 99 999$, 100,000$ - 119,999$, 120,000$ - 149,999$, 150,000$ or more, don’t know | Recoded into 3 categories (< 30,000, 30,000 < 99,000, ≥ 100,000) |
| Employed | About how many hours per week do you usually work at your job/business (paid or unpaid)? | (Number) hours per week | Yes, no |
| French spoken at home | What language do you speak most often at home? | English, French, English and French, Other (specify) | Yes, no |
| Born in Canada | Were you born…? | In Canada (name province), outside Canada (name country) | Yes, no |
| Mother university-educated | How much education has your mother had? | Did not finish high school, high school graduate, vocational, technical school, CEGEP, university, don't know, not applicable, other | Yes, no |
| *Lifestyle behaviours* |  |  |  |
| Smoked in the past year (Flynn et al. 1992) | Check the one box that describes you best… | I have never smoked a cigarette, even just a puff (in sc1-20 only), I have smoked cigarettes (even just a puff), but not at all in the past twelve months, I smoked cigarettes once or a couple of times in the past twelve months, I smoke cigarettes once or a couple of times each month, I smoke cigarettes once or a couple of times each week, I smoke cigarettes every day | Yes, no |
| Binge drank in past-year | In the past 12 months, how often did you drink 5 or more alcoholic beverages on one occasion | Never, less than once a month, 1-3 times per month, 1-6 times per week, every day | Used as continuous in the analysis |
| Weekly moderate and vigorous physical activity (Craig et al. 2003) | In the last 7 days, on how many days did you do moderate/vigorous physical activities (carrying light loads, bicycling at a regular pace, doubles tennis) for at least 10 minutes? Do not include walking.  On the days that you did moderate physical activities, how many minutes did you usually do per day? | None, (number) days in the last 7 days  (number) minutes per day | Variables were used to calculate meeting guidelines of ≥150 minutes of MVPA per week (yes, no) |
| Team sports in the past year | In the past 12 months, how many organized sports teams did you belong to (on which you practice with teammates or play against other teams)? | None, (number of teams) teams | Yes, no |
| Sleep quality | In general, how would you rate… the overall quality of your sleep at night? | Excellent, very good, good, fair, poor | Reverse coded and used as continuous in the analysis |
| *Eating behaviours* |  |  |  |
| Overeating frequency | How often do you overeat (eat more than usual, more than you wanted, more than you think is good for you)? | Never, rarely, sometimes, often, very often | Coded as continuous for analysis |
| Junk food score  Fruit and vegetable intake (Traynor et al. 2006) | How many times per day, per week or per month do you eat the following foods? (i) donuts or cakes or pastries, (ii) candy or chocolate bars, (iii) ice cream, (iv) potato chips, Fritos, Doritos, (v) soft drinks, (vi) fried chicken (Kentucky), (vii) hot dogs, (viii) hamburgers, (ix) french fries or poutine, (x) bacon or sausages, (xi) 100% fruit juices such as orange, grapefruit or tomato juice, (xii) fruit (not counting juice), (xiii) green salad, (xiv) potatoes (not including french-fries, fried potatoes or potato chips), (xv) carrots, (xvi) other vegetables (not counting carrots, potatoes or green salad) | (Number of times) times/day or (number of times) times/week or (number of times) times/month | Total fruits and vegetable per day  Junk food per day |
| Overeating compensation | Do you do any of the following to compensate for overeating during the 24 hours after overeating? (i) exercise or go for a walk, (ii) purge (vomit) or use laxatives, (iii) skip meals, (iv) give up on my diet for the rest of the day, (v) eat fruits, (vi) at vegetables, (vii) eat more high-protein foods, (viii) eat fewer fried foods, (ix) eat fewer sweets, (x) eat foods that are low in calories, (xi) eat more because I blew my diet for the day, (xii) do nothing differently | Never, rarely, sometimes, often, very often | Coded as continuous for analysis |
| *Weight-related indicators* |  |  |  |
| Weight | Measured | - | Used to calculate BMI |
| Height | Measured | - | Used to calculate BMI |
| Perceived weight | Do you consider yourself….? | Too thin, just about right, a little too heavy, much too heavy | Yes (a little too heavy, much too heavy), no (too thin, just about right) |
| Trying to lose weight (Rosen et al. 1987) | Currently, what are you doing about your weight? | I’m trying to lose weight, I’m trying to gain weight, I want to maintain my weight, I’m not doing anything about my weight | Yes, no (I’m trying to gain weight, I want to maintain my weight, I’m not doing anything about my weight) |
| *Psychological indicators* |  |  |  |
| Depression  symptoms (Major Depression Inventory scale) (Bech et al. 2001; Bech et al 1997) | In the past two weeks, how much of the time have you…? (i) felt low in spirits or sad, (ii) lost interest in, or could no longer enjoy your daily activities, (iii) felt lacking in energy and strength, (iv) felt less self-confident, (v) had a bad conscience or feelings of guilt, (vi) felt that life wasn’t worth living, (vii) had difficulty concentrating (when reading the newspaper or watching TV), (viii) felt very restless, (ix) felt subdued or slowed down, (x) had trouble sleeping at night or waking up too early, (xi) suffered from reduced appetite, (xii) suffered from increased appetite | At no time, some of the time, slightly less than half of the time, slightly more than half of the time, most of the time, all the time | Coded as continuous for analysis |
| Self-esteem (Rosenberg, 1965) | Please indicate your level of agreement with the following statements: (i) on the whole, I am satisfied with myself, (ii) at times, I think I am no good at all, (iii) I feel that I have a number of good qualities, (iv) I am able to do things as well as most other people, (v) I feel I do not have much to be proud of, (vi) I certainly feel useless at times, (vii) I feel that I’m a person of worth, at least on an equal place with others, (viii) I wish I could have more respect for myself, (ix) all in all, I am inclined to feel that I am a failure, (x) I take a positive attitude toward myself | Strongly agree, agree, disagree, strongly disagree | Coded as continuous for analysis |
| Stress in life | Thinking about the amount of stress in your life, would you say that most days are…? | Not at all stressful, not very stressful, a bit stressful, quite stressful, extremely stressful | Coded as continuous for analysis |
| *Body-related emotions* |  |  |  |
| Body-related envy and embarrassment  (Castonguay et al., 2014) | How often do you feel…? (i) Ashamed of the way you look, (ii) Proud that you are more attractive than others, (iii) Proud that you are a good-looking person, (iv) Inadequate when you think about your appearance, (v) Ashamed of your appearance, (vi) Proud of your superior appearance, (vii) Proud of the effort you place on maintaining your appearance, (viii) Proud of your efforts to improve the way you look, (ix) Proud that you have achieved your appearance goals, (x) Proud of your appearance efforts, (xi) Ashamed that you are a person who is unattractive, (xii) Guilty that you do not do enough to improve the way you look, (xiii) Guilty that you look the way you do, (xiv) Regret that you do not work on improving your appearance, (xv) Regret that you do not put effort into your appearance, (xvi) Proud that you are an attractive person, (xvii) Envious of another person’s appearance, (xviii) Inferior when you think about your appearance, (xix) Frustrated to see some people who have a great appearance with little effort, (xx) Unfair that some people have the “perfect” appearance, (xxi) Embarrassed about your appearance, (xxii) Foolish when your body and appearance are on display, (xxiii) Awkward when you are trying to improve your appearance, (xxiv) Nervous when you think about others seeing your appearance | Never, rarely, sometimes, often, always | Each of body-related authentic pride, body-related envy. body-related embarrassment and body-related hubristic pride was coded as continuous for analysis |
| Guilt and shame  (Conradt et al. 2007) | In the last 6 months, how often did you experience each of the following? (i) when I have eaten more than what I want, I experience feelings of guilt, (ii) when I am in a situation where others can see my body (pool, changing room), I feel ashamed, (iii) when I eat fattening food, I get distressed by the feeling that I did something wrong, (iv) the appearance of my body is embarrassing for me in front of others, (v) when I can’t manage to work out physically, I feel guilty, (vi) when I think of the possibility that others can see my naked body, I would rather hide somewhere, (vii) I am ashamed of myself when others get to know how much I really weigh, (viii) when I can’t get a grip on my weight, I blame myself, (ix) I blame myself when I break a good resolution concerning my eating, (x) I avoid exerting myself physically in front of others since I feel embarrassed, (xi) when I see myself in the mirror, I feel guilty and decide to do more for my figure, (xii) since the size of my clothes is embarrassing for me, I would rather avoid shopping for new clothes | Never, rarely, sometimes, often, always | Each of body-related shame and body-related guilt was coded as continuous for analysis |
| *Physical activity behaviour regulation* |  |  |  |
| Reasons for physical activity (Markland et al. 2004) | People engage in physical activity for many reasons. To what extent is each of the following is true for you? (i) I exercise because other people say I should, (ii) I feel guilty when I don’t exercise, (iii) I value the benefits of exercise, (iv) I exercise because it’s fun, (v) I don’t see why I should exercise, (vi) I take part in exercise because my friends/family/partner say I should, (vii) I feel ashamed when I miss an exercise session, (viii) it’s important to me to exercise regularly, (ix) I can’t see why I should bother exercising, (x) I enjoy my exercise sessions, (xi) I exercise because others will not be pleased with me if I don’t, (xii) I don’t see the point of exercising, (xiii) I feel like a failure when I haven’t exercised for a while, (xiv) I think it’s important to make the effort to exercise regularly, (xv) I find exercise a pleasurable activity, (xvi) I feel under pressure from my friends and family to exercise, (xvii) I get restless if I don’t exercise regularly, (xviii) I get pleasure and satisfaction from participating in exercise, (xix) I think exercising is a waste of time | Not true for me, rarely true for me, sometimes true for me, often true for me, very often true for me | Each of amotivation. external, introjected, identified and intrinsic motivation was coded as continuous for analysis |
| Variety of exercise behaviors | How true are each of the following for you? When I exercise, I feel like… (i) I engage in a variety of exercises, (ii) I try a range of exercises, (iii) I change the type of exercise that I do, (iv) My exercise program is varied, (v) I experience variety in my exercise | False, mostly false, more false than true, more true than false, mostly true, true | A continuous score was created for analysis |
| *Social and societal indicators* |  |  |  |
| Weight commentary  (adapted from Verbal Commentary on Physical Appearance Scale (Herbozo & Thompson, 2006) | In the past 2 years, how often did people in your life…? (i) make negative comments about your weight, (ii) encourage you to lose weight, (iii) encourage you to gain weight | Never, rarely, sometimes, often, always | Each of pressure to lose weight, pressure to gain weight, experience negative weight comments was coded as continuous for analysis |
| Friends encourage exercising (adapted from Peer Norms Scale: Ling et al. 2014) | Indicate your level of agreement with the following. My close friends … (i) encourage me to be physically active, (ii) would disapprove if they saw me just sitting around, (iii) think i should exercise most days of the week, (iv) think i should be physically active | Strongly disagree, disagree, neither agree nor disagree, agree, strongly agree | A continuous score was created for analysis |
| *Health-related indicators* |  |  |  |
| Health (from CCHS)  (Gravel & Beland, 2005) | In general, how would you rate…? (i) your health, (ii) your mental health | Excellent, very good, good, fair, poor | Reverse coded. A continuous score was created for health and mental health for analysis |
| Diagnoses | Has a health professional ever diagnosed that you have any of the following? (i) mood disorder (depression, bipolar disorder); (ii) anxiety disorder (phobia, fear of social situations, obsessive-compulsive disorder, panic disorder, generalized anxiety disorder); (iii) eating disorder (anorexia, bulimia) | No, yes | No, yes |

# References

# Flynn BS, Worden JK, Secker-Walker RH, Badger G J, Geller BM, Costanza MC. Prevention of cigarette smoking through mass media intervention and school programs. Am J Public Health 1992;82:827–34

# Craig CL, Marshall AL, Sjostrom M et al. International physical activity questionnaire: 12-country reliability and validity. Med Sci Sports Exerc 2003;35:1381-95

# Traynor MM, Holowaty PH, Reid DJ, Gray-Donald K. Vegetable and fruit food frequency questionnaire serves as a proxy for quantified intake. Can J Public Health 2006;97:286-90

Rosen, J.C., 1987. The validity of self-reported weight loss and weight gain efforts in adolescents. Int. J. Eat. Disord. 6, 515–523

Bech P, Rasmussen NA, Olsen LR, Noerholm V, Abildgaard W. The sensitivity and specificity of the Major Depression Inventory, using the Present State Examination as the index of diagnostic validity. J Affect Disord 2001;66:159-64.

Bech P, Stage KB, Nair NP, Larsen JK, Kragh-Sorensen P, Gjerris A. The Major Depression Rating Scale (MDS). Inter-rater reliability and validity across different settings in randomized moclobemide trials. Danish University Antidepressant Group. J Affect Disord 1997;42:39-48.

Gravel R, Béland Y. The Canadian Community Health Survey: mental health and well-being. The Canadian Journal of Psychiatry. 2005 Sep;50(10):573-9.

Rosenberg M. Society and the adolescent self-image. Princeton, NJ: Princeton University Press; 1965

Castonguay AL, Sabiston CM, Crocker PR, Mack DE. Development and validation of the body and appearance self-conscious emotions scale (BASES). Body Image. 2014 Mar 1;11(2):126-36.

Conradt M, Dierk JM, Schlumberger P, Rauh E, Hebebrand J, Rief W. Development of the Weight- and Body-Related Shame and Guilt scale (WEB-SG) in a nonclinical sample of obese individuals. J Pers Assess 2007;88:317-27

Markland D, Tobin V. A modification to behavioural regulation in exercise questionnaire to include an assessment of amotivation. J Sport Exerc Psychol 2004;26:191-6

Herbozo S, Thompson JK. Appearance-related commentary, body image, and self-esteem: Does the distress associated with the commentary matter?. Body image. 2006 Sep 1;3(3):255-62.

Ling, J., Robbins, L. B., Resnicow, K., & Bakhoya, M. (2014). Social support and peer norms scales for physical activity in adolescents. American journal of health behavior, 38(6), 881-889. doi:10.5993/AJHB.38.6.10)

| **Table S4**. Number and percent of missing data for each study variable (n=676), XXXX study 2010-20 | | |
| --- | --- | --- |
|  | n | % |
| *Sociodemographic indicators* |  |  |
| Age | 0 | 0.0 |
| Sex | 0 | 0.0 |
| Participant attended university | 3 | 0.4 |
| Household income | 34 | 5.0 |
| Employed | 7 | 1.0 |
| *Lifestyle behaviours* |  |  |
| Smoked cigarettes in past 12-months | 0 | 0.0 |
| Binge drank in past-year | 7 | 1.0 |
| Meets MVPA guidelines | 14 | 2.1 |
| Team sports in past year | 6 | 0.8 |
| Sleep quality | 2 | 0.3 |
| *Eating behaviours* |  |  |
| Often overeat | 21 | 3.1 |
| Total fruits and vegetable per day | 48 | 7.1 |
| Junk food per day | 57 | 8.4 |
| Compensatory behaviors after overeating | 6 | 0.8 |
| *Weight-related indicators* |  |  |
| BMI | 97 | 14.0 |
| Self-report overweight | 2 | 0.3 |
| Trying to lose weight | 5 | 0.7 |
| *Psychological indicators* |  |  |
| Depressive symptoms | 15 | 2.2 |
| Self-esteem | 3 | 0.4 |
| Daily stress | 2 | 0.3 |
| *Body-related emotions* |  |  |
| Body-related shame | 47 | 6.9 |
| Body-related guilt | 47 | 6.9 |
| Body-related authentic pride | 47 | 6.9 |
| Body-related envy | 47 | 6.9 |
| Body-related embarrassment | 47 | 6.9 |
| Body-related hubristic pride | 47 | 6.9 |
| *Physical activity behaviour regulation* |  |  |
| Amotivation | 46 | 6.8 |
| External | 46 | 6.8 |
| Introjected | 46 | 6.8 |
| Identified | 46 | 6.8 |
| Intrinsic | 46 | 6.8 |
| Variety of exercise behaviors | 5 | 0.7 |
| *Social and societal indicators* |  |  |
| Pressure to engage in PA | 5 | 0.7 |
| Pressure to lose weight | 6 | 0.8 |
| Pressure to gain weight | 5 | 0.7 |
| Experience negative weight comments | 6 | 0.8 |
| *Health-related indicators* |  |  |
| Self-rated health | 3 | 0.4 |
| Self-rated mental health | 4 | 0.5 |
| Diagnosed anxiety disorder | 46 | 6.8 |
| Diagnosed mood disorder | 46 | 6.8 |
| Diagnosed eating disorder | 46 | 6.8 |
